# Developmental Trajectory of Theory-of-Mind Decline in Older Adults

**DOI:** 10.1093/geroni/igaa057.1167

**Published:** 2020-12-16

**Authors:** W Quin Yow, Xiaoqian Li, Jiawen Lee

**Affiliations:** Singapore University of Technology & Design, Singapore, Singapore

## Abstract

Theory-of-Mind (ToM) is critical to individual social competence and mental health across the lifespan (Frith, 2008). Though it is often discussed as one broad construct, ToM abilities can be viewed as following a developmental trajectory: from early emotion recognition and gaze following to more advanced inferences about others’ beliefs, perspectives, and intentions (Hutchins et al., 2012). Despite current literature suggesting that ToM abilities may be impaired in late adulthood, there is no consensus regarding whether ToM abilities are differentially affected by age. In this study, we examined younger adults (N=18, aged 19-30) and older adults (N=13, aged 58-76) on their ToM competence across three levels of ToM abilities: Early-ToM (e.g., recognizing a happy face), Basic-ToM (e.g., perspective-taking and false-belief reasoning), and Advanced-ToM (e.g., inferring second-order emotion and false belief). All participants completed a Theory-of-Mind Task Battery consisting of three subscales that assessed the three levels of ToM, where participants viewed vignettes and answered questions about the protagonists’ feelings and beliefs. Overall, younger adults outperformed older adults on the battery, F(1,29)=7.34, p=.011. However, a significant interaction between age and ToM levels (p=.010) revealed that Early and Advanced ToM (ps>.25) were not as affected by age as Basic ToM (p=.007). Older adults have difficulty in inferring others’ perspectives/beliefs while their attributions of emotion and higher-order false beliefs are relatively preserved compared to the younger adults. These findings provide important insights into the impact of age on various levels of ToM and could help inform early detection of ToM decline in normal aging.

